# Human pursuance of equality hinges on mental processes of projecting oneself into the perspectives of others and into future situations

**DOI:** 10.1038/s41598-017-05469-9

**Published:** 2017-07-19

**Authors:** Hirofumi Takesue, Carlos Makoto Miyauchi, Shiro Sakaiya, Hongwei Fan, Tetsuya Matsuda, Junko Kato

**Affiliations:** 1 Graduate School of Law and Politics, The University of Tokyo, Bunkyo, Tokyo, Japan; 2 Institute for Diversity and Adaptation of Human Mind, The University of Tokyo, Bunkyo, Tokyo, Japan; 3 Tamagawa University Brain Science Institute, 6-1-1 Tamagawagakuen, Machida, Tokyo 194-8610 Japan; 4 Graduate School of Social Sciences, Tokyo Metropolitan University, Hachioji, Tokyo, Japan; 5 Oracle (China) Software Systems Co., Ltd., 21/F, Unit C, Yuanyang Guanghua Center, No.5, Jinghua (S) St.,C Beijing, Beijing, 100020 China

## Abstract

In the pursuance of equality, behavioural scientists disagree about distinct motivators, that is, consideration of others and prospective calculation for oneself. However, accumulating data suggest that these motivators may share a common process in the brain whereby perspectives and events that did not arise in the immediate environment are conceived. To examine this, we devised a game imitating a real decision-making situation regarding redistribution among income classes in a welfare state. The neural correlates of redistributive decisions were examined under contrasting conditions, with and without uncertainty, which affects support for equality in society. The dorsal anterior cingulate cortex (dACC) and the caudate nucleus were activated by equality decisions with uncertainty but by selfless decisions without uncertainty. Activation was also correlated with subjective values. Activation in both the dACC and the caudate nucleus was associated with the attitude to prefer accordance with others, whereas activation in the caudate nucleus reflected that the expected reward involved the prospective calculation of relative income. The neural correlates suggest that consideration of others and prospective calculation for oneself may underlie the support for equality. Projecting oneself into the perspective of others and into prospective future situations may underpin the pursuance of equality.

## Introduction

Equality is one of the greatest concerns in the humanities and social sciences. Philosophers and social scientists concur that selfishness is constrained by uncertainty about one’s social position, i.e., the veil of ignorance (VoI), but disagree on what motivates humans to pursue equality^1^. Political theorists contend that humans support equality because they wish to be *fair*; thus, when social status, abilities, and other characteristics are unknown, i.e., behind the VoI, a *consideration of others* prioritizes the welfare of the worst-off individuals^2^. In contrast, economic theorists focus on the *prospective calculation for oneself*; thus, their utilitarian view posits that equality is chosen behind the VoI if *risk aversion* motivates people to maximize their worst foreseen pay-off^3, 4^. Different motivations have distinct social consequences with a bearing on whether equality is or is not likely to be achieved^1, 5^ in society^3, 4^. Specifically, equality in income redistribution has been one of the greatest concerns in our society. Therefore, scholarly interest has often focused on whether the uncertainty associated with the VoI increases the possibility of redistributive equality and, if so, what motivates people to support it in a real society^1, 2^.

Behavioural studies have examined whether a distributive decision with uncertainty was motivated by consideration of others or a prospective calculation of self-risk but found mixed results without obtaining data on the psychological process^6–8^. Neuroscience experiments have explored egalitarian preferences^9^ and compared the equality of self and equality among others^10, 11^ and the equality of opportunity and equality of outcomes^12^ but have not examined the decision on distributive equality. A recent study reported that a third-party judgement to distribute to the poor was associated with an individual choice of the safe lottery when selective attention to the worst-off (gambling/distribution) outcomes activated the right temporoparietal junction (rTPJ)^13^. The neural correlates of a decision on distributive equality have not yet been examined, but studies have thus far suggested that both consideration of others and self-risk calculation might be implicated in the human pursuance of equality. Actually, accumulating data have begun to suggest that if examined in a social context, consideration of others and the prospective calculation for oneself may share a core process of projecting oneself into social situations beyond those that arise in the immediate environment^14, 15^. Building on the literature, this study directly examined the neural correlates of equality decisions and explored the core neural process associated with the human pursuance of equality.

**Figure 1:**
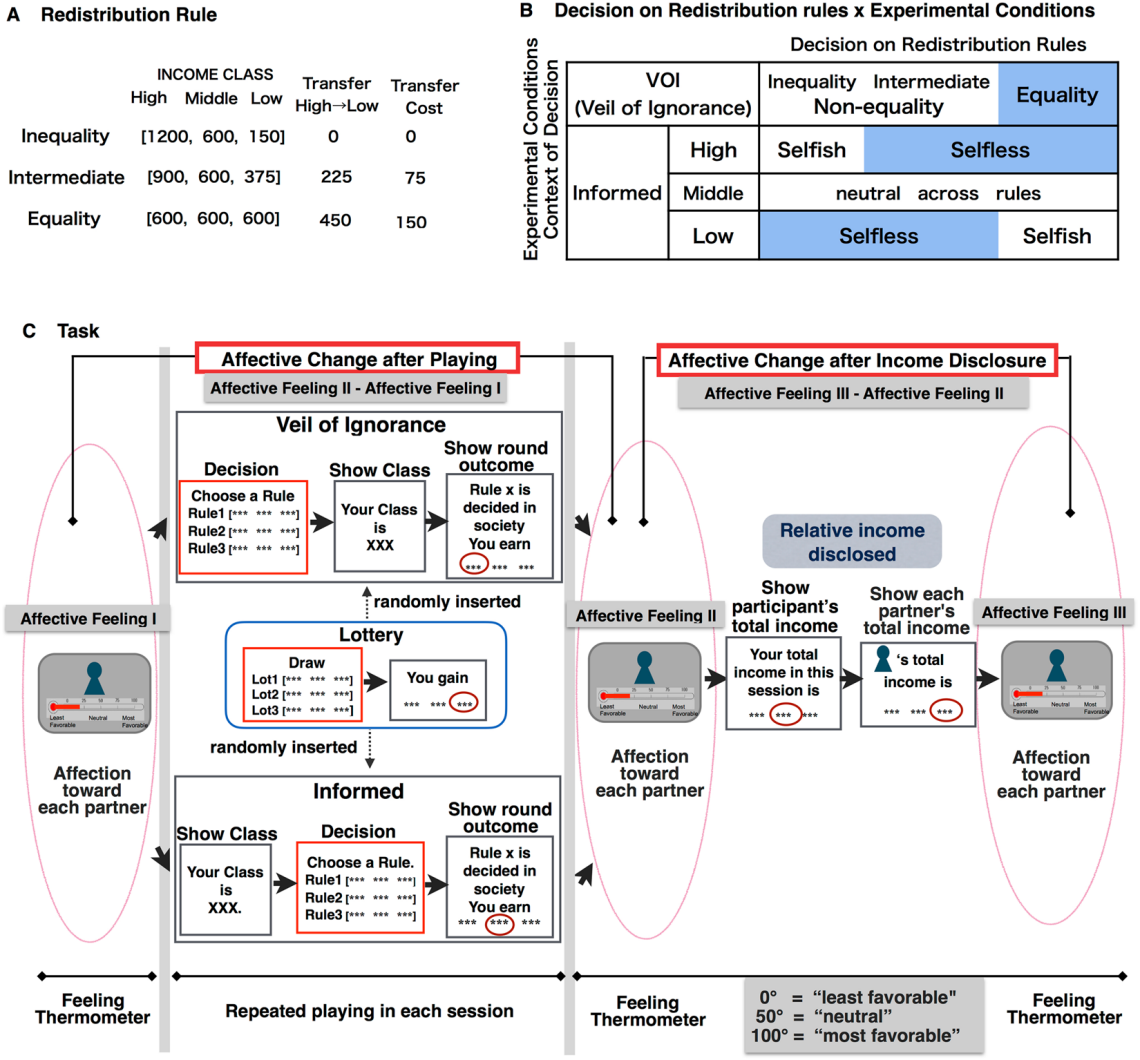
Experimental Design. (**A**) Each participant was required to choose one of three redistribution rules: preserving the status quo of inequality, advancing intermediate redistribution, or achieving absolute equality. A total of 25% of the transferred income was lost as a cost. The equality rule incurred the largest cost, whereas the inequality rule was cost-free. During the games, these rules were termed Rules 1, 2, and 3, respectively, to avoid generating a decision bias. (**B**) When making a decision behind the VoI, class did not matter; thus, the rule was chosen in terms of equality. When the classes were “informed”, the experimental conditions were distinguished in terms of high-, middle-, and low-income classes. Therefore, we distinguished the decisions as an equality or non-equality decision in the VoI and a selfless or selfish decision in high- and low-income classes, whereas decisions were neutral in the middle class. (**C**) On repeated runs of each session (the Informed and VoI conditions), the participant chose a rule (red-boxed screen) and was shown the payoff that corresponded to her assigned class along with the rule chosen at the society level. Earnings from each round accumulated to a total income that was revealed at the end of game. Participants were told that the total income determined the payment for completion of the experiment. Before and after playing each session, participants were shown a photo of each partner and were asked to rate how much they (dis)liked each partner on a thermometric scale, i.e., a feeling thermometer (Affective Feeling I and II, respectively). The change in affection after playing was calculated by subtracting Affective Feeling I from Affective Feeling II. Next, we disclosed their own and their partners’ incomes over the entire session and asked the participant to again rate their feelings for their partners. We subtracted the rating immediately after playing (Affective Feeling II) from that after the disclosure of income (Affective Feeling III) and used it as a measure, namely, the change in affection after income disclosure. The relative income was calculated by dividing the participants’ total income by the partners’ income. In each session, a lottery game with the same format was randomly inserted. The redistribution-specific activation was calculated by subtracting the activation evident when drawing the lottery from that observed upon a redistributive decision.

Our functional magnetic resonance imaging (fMRI) study used an experimental framework with a relevance to a real problem, i.e., equality in income redistribution. The experimental game used here imitates a real problem of redistribution among income classes, replicating the uncertainty of the VoI that has been considered to affect support for equality in society. Before the experiment, the participant was told that she would belong to a hypothetical society that consisted of three income classes (high, middle, and low) with the same number of people in each class. The participant voted on one of three redistribution rules: *inequality*, *intermediate* redistribution, or absolute *equality* (Fig. 1A) under contrasting conditions with and without uncertainty: the participant either did not know (VoI) or did know (Informed) which class was assigned to her (high, middle, and low; Fig. 1B). During repeated rounds within a game, each of the three classes was randomly assigned to three players (participant and two others) from one round to the next, and each player earned income associated with her class along with the redistribution rule chosen by a majority vote (Fig. 1C). To examine the neural correlate specific to the redistributive decision, we randomly inserted the lottery game and focused on the contrast after subtracting the activation associated with the lottery decision from the activation associated with the distributive decision-making (Fig. 1C; red-boxed screen).

The results indicate the presence of a core mental process that underpinned the apparently disparate motivators for equality, i.e., *consideration of others* and *prospective calculation for oneself*. The dorsal anterior cingulate cortex (dACC) and the caudate nucleus were activated by an equality decision behind the VoI but by selfless decisions when the participants were informed of high and low income classes at the time of the decision (Fig. 1B). We inferred the mental processes by analysing two quantifiable measures, affective changes and expected reward, based on post-session observations that were independent of brain activation during the decision-making stage (Fig. 1C, see Methods)^16, 17^. Activation correlated with affective changes indicates that the dACC and caudate nucleus were associated with the attitude to prefer accordance over non-accordance with others. Furthermore, when an equality decision was made behind the VoI, stronger activation in the caudate nucleus was found among those who were to earn smaller amounts of total income than their partners. Since the total income had yet to be realized during game play, the participants only expected it while making a decision. The caudate activity during decision-making may represent the expectation of lower income than that of others and thus be associated with the prospective calculation of a reward.

Together, the neural correlates indicate that both consideration of others and self-prospection, which appear as distinct motivators in behaviours, may influence equality decisions in social contexts. When equality is pursued under conditions of uncertainty in society, individuals may view the world from the perspective of others or simulate their own future situation. The present study investigated the neural correlates of equality decisions in our society and explored the mental projection process that may underlie the *consideration of others* and *prospective calculation for oneself* in the social context.

## Results

### Behavioural analysis

#### Decisions

In our experiment, which imitated a real redistribution situation in society, an equality rule would incur the largest transfer cost, but an inequality rule would incur no transfer cost and would thus preserve the overall payoffs in society (see Fig. 1A and Methods). In an equality decision behind the VoI, therefore, participants were required to accept the transfer cost for an equal outcome. In the low income condition, the choice of selfless rules (inequality and intermediate rules) is considered a decision to prioritize the overall payoff to society over one’s own payoff. In the high income condition, the choice of the selfless rules (equality and intermediate rules) is considered a decision to prioritize equality in society over one’s own payoff.

**Figure 2:**
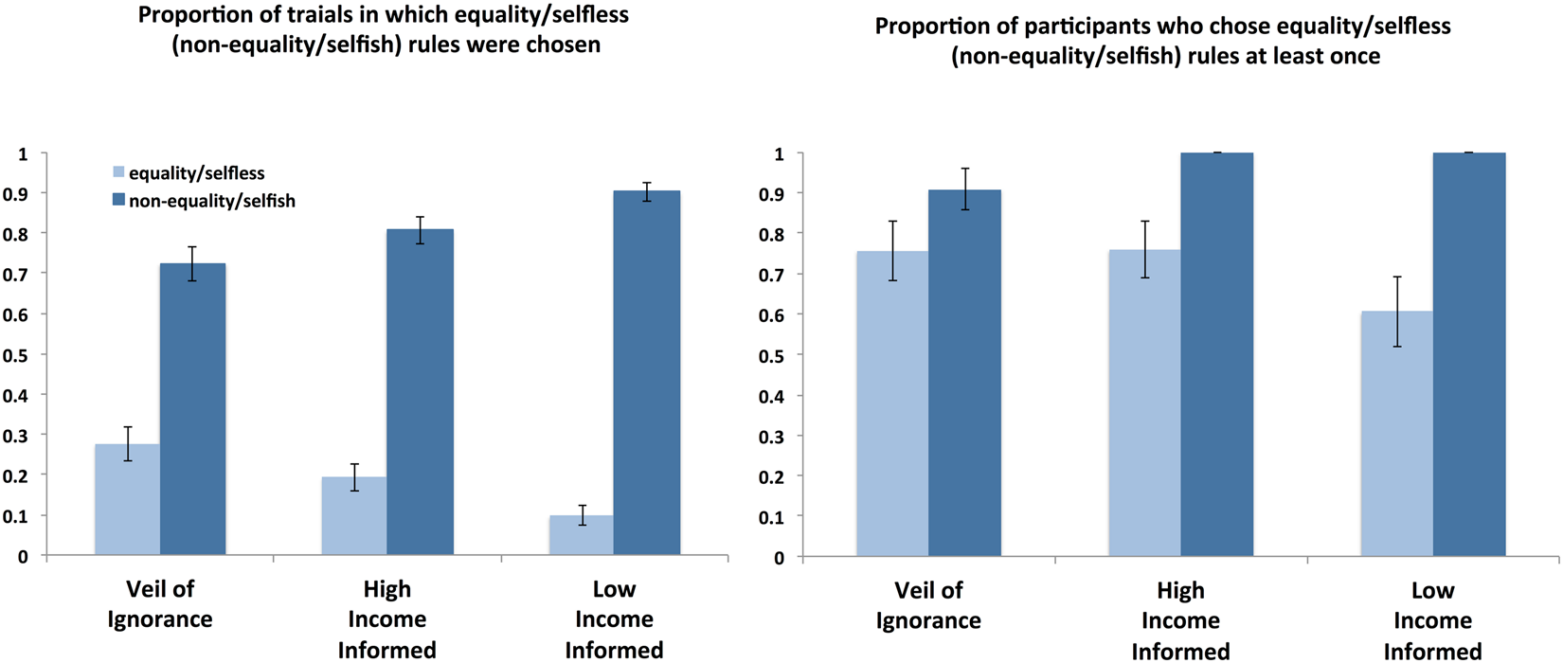
Behavioural analysis of decisions on redistribution rules. In the VoI condition, decisions on the equality rule were compared with those of non-equality (intermediate and inequality) rules. In the Informed condition, selfish decisions on inequality (equality) rules in high (low) classes were compared with selfless decisions, i.e., equality and intermediate (inequality and intermediate) rules. Data are expressed as mean ± s.e.m. Panel A: Proportion of trials in which equality/selfless (non-equality/selfish) rules were chosen in the VoI, High Income (Informed), and Low Income (Informed) conditions. Panel B: Proportion of participants who chose equality/selfless (non-equality/selfish) rules at least once in the VoI, High Income (Informed), and Low Income (Informed) conditions.

Building on this understanding, we summarized the results focusing on the VoI and the high and low income conditions. Figure 2A shows the proportion of instances (averaged across the participants) in which each of the rules was chosen. In the VoI condition, we compared the proportion of trials in which the equality and non-equality rules were chosen and found that the propensity was towards the non-equality rule. Non-equality rule was chosen in 72.3% ± 2.2% of trials whereas the equality rule was chosen in 27.7% ± 2.2% of the trials. When the participant was informed that she was in the high (low) class, the inequality (equality) rule was consistent with the participant’s self-interest; thus, we compared the proportion of instances in which these rules were chosen with those in which the selfless rules were chosen, i.e., equality and intermediate (inequality and intermediate) rules. Selfish rules were chosen in 80.7% ± 1.7% and 90.2% ± 1.2% of the trials in the high- and low-income classes, respectively, whereas the selfless rules were chosen in 19.3% ± 1.7% and 9.8% ± 1.2% of the trials, respectively. The proportion of non-equality/selfish rules was significantly higher than that of the equality/selfless rules in the VoI/Informed conditions. Self-interested behaviour was salient when the income classes were informed, which is consistent with the recent findings in the field of social psychology^18, 19^.

Second, we examined the proportion of participants who chose the equality/selfless rules at least once. The proportions of participants who ever chose the selfless rules were 76.0% ± 3.6% and 60.9% ± 4.3% in the high and low classes, respectively, whereas 75.7% ± 3.7% of the participants chose the equality rule at least once behind the VoI (Fig. 2B). All the participants ever chose the selfish rules in the high and low classes and 90.9% ± 2.6% of the participants chose the non-equality rules at least once behind the VoI. In the Informed conditions, as a result, the proportion of participants who ever chose the selfish rules was significantly higher than the proportion of participants who ever chose the selfless rules, but in the VoI condition, the proportions of those who ever chose the equality and non-equality rules were not significantly different.

Taken together, in the Informed (VoI) conditions, the participants’ decision leaned towards the selfish (non-equality) rules, but a majority of participants chose, though much less frequently, the selfless (equality) rules at least once (for the entire results, see Supplementary Table 1).

#### Contrasting changes in affective feelings underline the distinct attitude to be in accordance or not in accordance with others

We asked participants to rate their affective feeling towards partners three times during each session (Affective Feelings I, II, and III) and calculated the affective changes (Fig. 1C, see Methods). We investigated the participant’s attitude to examine the effect of the material vantage on affective change before and after the total income of all (the participant and two partners) was disclosed (*change in affection after income disclosure*: Affective Feelings III–II). If the material vantage affected their feeling, we expected, for example, that participants would disfavour partners when their income was disclosed to be lower but not higher than theirs. However, when a participant’s relative income level was higher than their partners’, the response to income disclosure was classified into either a decrease or an increase in the affective ratings. Here, a decrease in an affective rating did not result from an ill feeling incurred by the others’ advantage and was thus unexpected from the material vantage. Instead, a decrease in the rating may plausibly imply that participants experienced a decreased affection towards their partners since the disclosure of the income (regardless of one’s own material vantage) made them aware of the competition with partners.

**Figure 3:**
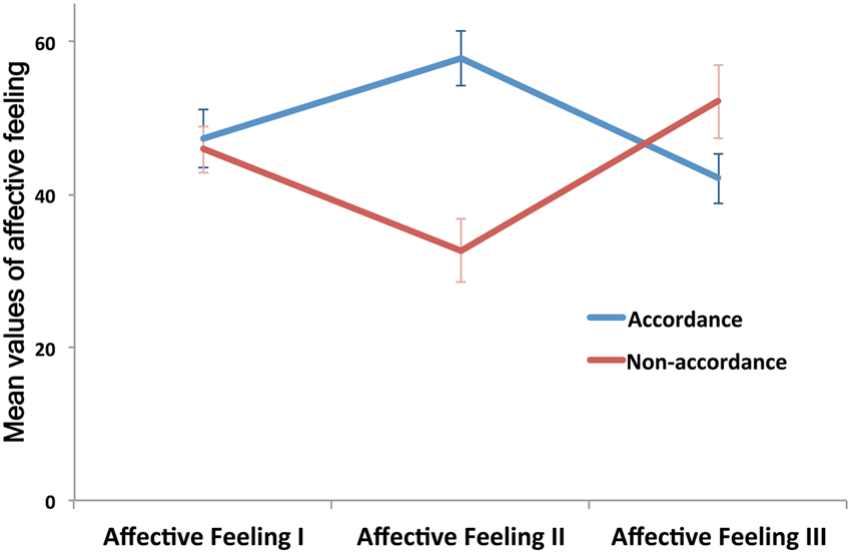
Contrasting changes in affective feelings underline the distinct attitude to be in accordance or non-accordance with others. To control for the effect of material vantage on the affective feeling, we focused on the cases in which a participant’s income was higher than their partners’ income. We distinguished two cases with changes in the affective rating that were unexpected and expected from the material vantage. Based on the comparison of the contrasting pattern, i.e., going up (down) and up (down), in the ratings of Affective Feelings I, II, and III, we regarded the participants as preferring *accordance* and *non*-*accordance* with others, respectively. The transformation of the affective change reveals a significant difference for the *accordance* and *non*-*accordance* attitudes. Before playing the game, their ratings (Affective Feeling I) were not significantly different (*p* = 0.783, two-tailed *t*-test; 47.3 ± 3.8 and 45.9 ± 3.1), but after playing, their ratings (Affective Feeling II) were significantly different (*p* < 0.001, two-tailed *t*-test; 57.7 ± 3.6 and 32.6 ± 4.2) for the *accordance* and *non*-*accordance* attitudes. In particular, for the *non*-*accordance* attitude, the decrease in ratings during play was significant (−13.3, *p* < 0.001). However, the ratings after income disclosure (Affective Feeling III) were reversed, i.e., they decreased to 42.1 ± 3.3 and increased to 52.2 ± 4.8 for the *accordance* and *non*-*accordance* attitudes, respectively. Data are expressed as mean ± standard error.

We further explored the results from situations in which the participants earned a higher income than their partners. We distinguished and compared those that showed a decrease (increase) in affection that was unexpected (expected) from the material vantage. When we followed the transitional change from Affective Ratings I and II, to III, a contrasting pattern of going up (down) and down (up) was observed, respectively (Fig. 3). A decrease (increase) in the affective rating after income disclosure (Affective Feeling III – Affective Feeling II) followed an increase (decrease) in the affective rating after playing (Affective Feeling II – Affective Feeling I). Note that the contrasting pattern of changes was not an artefact that coincidentally resulted from extreme ratings. Affective Feeling I was almost the same between the cases when affective changes subsequently revealed the contrasting pattern (*p* = 0.783, two-tailed *t*-test; 47.3 ± 3.8 and 45.9 ± 3.1). Affective Feeling II was significantly different between two cases (*p* < 0.001, two-tailed *t*-test; 57.7 ± 3.6 and 32.6 ± 4.2) but neither of these values was too high (low) to prevent a further increase (decrease) in Affective Feeling III. However, Affective Feeling III was reversed, i.e., the former decreased to 42.1 ± 3.3 and the latter increased to 52.2 ± 4.8.

Taken together, the contrasting pattern, i.e., going up (down) and down (up), of the affective ratings represents the distinct attitude towards others. In the former pattern of change, the affective rating towards their partners increased during game play but reversed (decreased) when the disclosure of the income revealed the participant’s competitive relationship with the partners. Since an affective change was unexpected from the material vantage, we regarded this attitude as one that leans towards *accordance* with others. We then distinguished it from the *non*-*accordance* attitude in which the participants exhibited a reversal of the affective rating. Since we excluded the cases when participants revealed no changes in their affective ratings after the disclosure of a higher income than partners’, we observed nineteen cases in *accordance* and thirty-one cases in *non*-*accordance* (see Methods for details).

Female participants more frequently revealed the *accordance* attitude (six out of nineteen cases) than the *non*-*accordance* attitude (two out of thirty-one cases), but the gender difference in attitude was not significant (*p* = 0.087, chi-squared test). We also did not find a significant difference in the age of the participants who revealed the *accordance* (21.1 ± 2.5) and *non*-*accordance* (21.9 ± 2.4) attitudes.

#### Correlations between affective changes and material gains

Since affective changes were plausibly influenced by material vantage, we examined whether affective changes after playing and income disclosure were correlated with participants’ total incomes. We defined the participant’s total income compared with that of their partners as the relative income (participant’s total income/partners’ total income; see Methods), in addition to the absolute income that simply represents the absolute value of the participant’s total income. Neither the affective change after playing nor the affective change after income disclosure was correlated with the absolute income. In contrast, the relative income was correlated with the affective change after income disclosure (*r* = −0.23, *p* < 0.001).

### Neural analysis

#### The caudate nucleus and dACC were activated by equality decisions behind the VoI and by selfless decisions in both high- and low-income classes

**Figure 4:**
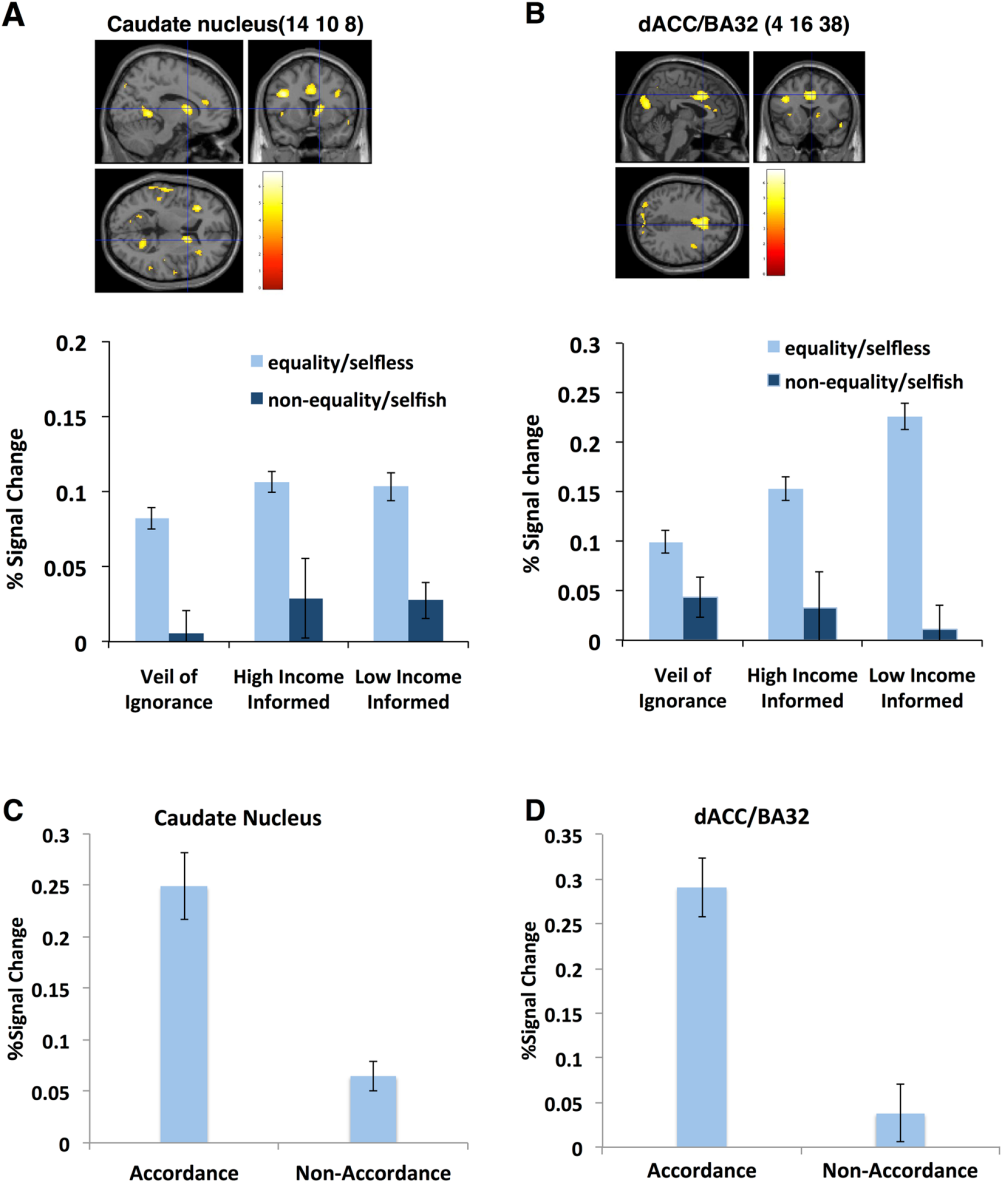
Panel A, B: Activation associated with an equality decision in the VoI condition but with a selfless decision in the High- and Low-income conditions. The bar plots represent the percent signal change (mean ± standard error) of the peak coordinate. Stronger activation [family-wise error (FWE)-corrected *p* < 0.05 for multiple comparisons across the whole brain] was associated with equality rather than non-equality decisions in the VoI condition but with selfless rather than selfish decisions when participants were informed of their high- and low-income classes, respectively. The caudate nucleus (14, 10, 8): FWE-corrected *p* = 0.029 for multiple comparisons across the whole brain, *k* = 13; the dorsal anterior cingulate cortex (dACC) (4, 16, 38), FWE-corrected p = 0.006 for multiple comparisons across the whole brain, *k* = 116. Neural data were obtained from the contrast in which the activation evident when drawing the lottery was subtracted from that observed upon a redistributive decision. Panel C, D: Activation in the caudate nucleus and dACC was associated with the attitude to be in accordance with others. Differential activation along with affective changes was observed only when participants had higher rather than lower income than their partners. Activation was significantly stronger in both regions (*p* < 0.05, two-tailed *t*-test) when participants revealed the attitude to be in accordance with others, i.e., decreased affection after income disclosure towards their partners whom they came to favour during the game. The bar plots represent the percent signal change (mean ± standard error) of the peak coordinate. Neural data were obtained from the contrast in which the activation evident when drawing the lottery was subtracted from that observed upon a redistributive decision.

The present study focused on redistribution-specific brain activation that controlled the effect of risk calculation of individual gain and reflected a decision that specifically involved distribution in a social context (see Methods). We applied the family-wise error (FWE)-corrected threshold of *p* < 0.05 and found activation in two regions that differed between rule decisions. When participants made equality decisions behind the VoI, the caudate nucleus (*x* = 14, *y* = 10, *z* = 8; FWE-corrected *p* = 0.029 for multiple comparisons across the whole brain) and the dACC (*x* = 4, *y* = 16, *z* = 38; FWE-corrected *p* = 0.006 for multiple comparisons across the whole brain) were activated (Table 1, Supplementary Table 3 for all of the ANOVA data and Supplementary Fig. 1). In contrast, when the participants were informed of the income classes at the time of the decision, these regions were activated when participants made selfless decisions (i.e., chose rules that were against their own interest, namely, more equal rules for those with high income and less equal rules for those with low income). In summary, stronger activation of the caudate nucleus and the dACC (% signal changes) was observed with equality decisions behind the VoI and with selfless decisions for high- and low-income classes (Fig. 4A and B, respectively). We did not detect regions above threshold in the opposite contrast, i.e., when participants made a non-equality/selfish decision.

**Table 1:** Positive interaction: Equality/Selfless > Non-Equality/Selfish.

	MNI coordinates			*k*	FWE-corrected *p*	*F* (1, 444)	*Z*
	*x*	*y*	*z*				
Caudate nucleus	14	10	8	13	0.029	4.47	4.41
dACC BA32	4	16	38	116	0.006	4.87	4.80

FWE-corrected *p* < 0.05 for multiple comparisons across the whole brain.

The number of *k* on the FWE-corrected *p* < 0.05 *t*-map.

Selfless = High (Intermediate/Equality): Low (Equality/Intermediate).

Selfish = High (Inequality): Low (Equality).

#### Stronger activation in the caudate nucleus and dACC was associated with the attitude of accordance with others

We further examined the activation in the caudate nucleus and dACC that was associated with the selfless/equality decision. More specifically, we compared activation in the caudate nucleus and dACC when the contrasting changes in the affective rating revealed the distinct attitudes of *accordance* or *non*-*accordance* with others (see Behavioural Analysis and Fig. 3). Activation was significantly stronger in both regions (two-tailed *t*-test, *p* < 0.05) in participants with the *accordance* attitude than in those with the *non*-*accordance* attitude (Fig. 4C and D, respectively). Differential activation along with affective changes was found when the participants’ income was higher than the partner (as presented in Fig. 4) but not when the participants’ income was lower than the partner.

#### The caudate nucleus was activated by an equality decision independent of the VoI

**Figure 5:**
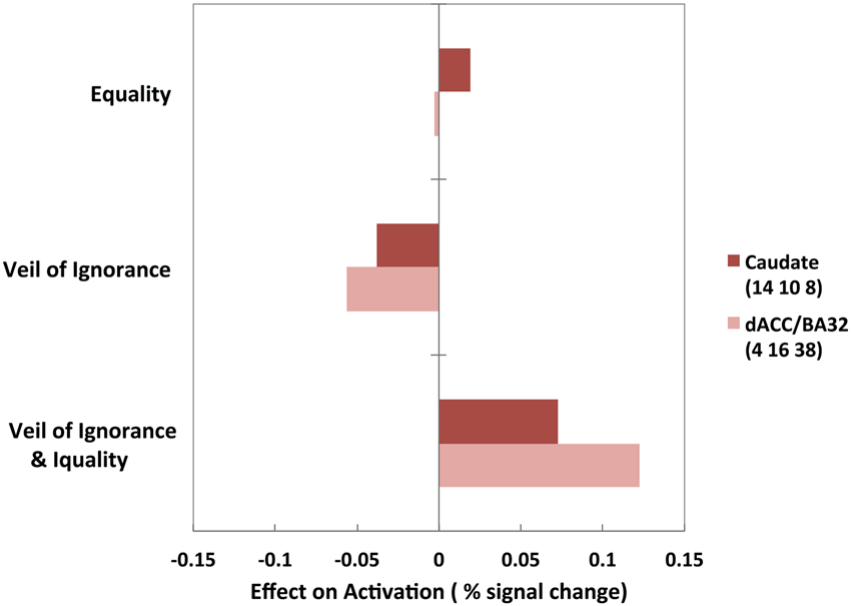
The caudate nucleus was activated by an equality decision, but the dACC activation depended on the presence of the VoI. An equality decision increased the activation of only the caudate nucleus and thus reduced activation of the dACC. The VoI condition reduced the activation of both brain regions but more so that of the dACC than the caudate nucleus. When the effects of the two factors (an equality decision and the VoI) were combined (i.e., when an equality decision was made behind the VoI), activation of the dACC was greater than that of the caudate nucleus. Caudate nucleus: 4, 10, 8; FWE-corrected *p* = 0.029 for multiple comparisons across the whole brain; *k* = 13. dACC: 4, 16, 38; FWE-corrected *p* = 0.006 for multiple comparisons across the whole brain; *k* = 116.

The caudate nucleus but not the dACC was activated by equality decisions regardless of the presence of the VoI. We decomposed the combined effects of the two factors (the VoI and an equality decision: Fig. 5) by calculating the invariant effects of each factor in terms of the activation of the two regions (see Methods and Supplementary Information). Compared with activation associated with the lottery decision, an equality decision increased activation only in the caudate nucleus and reduced activation in the dACC. The VoI condition reduced the activation of both regions but affected the dACC to a greater extent than the caudate nucleus. When an equality decision was made behind the VoI, the dACC was activated to a greater extent than the caudate nucleus (see Supplementary Fig. 2 for detailed results). These findings indicate that the caudate nucleus was activated by an equality decision alone independent of the VoI, whereas the neural process for a decision favouring equality may have been fundamentally reshaped by the VoI in the dACC.

#### Activation in the caudate nucleus was correlated with an expected reward

**Figure 6:**
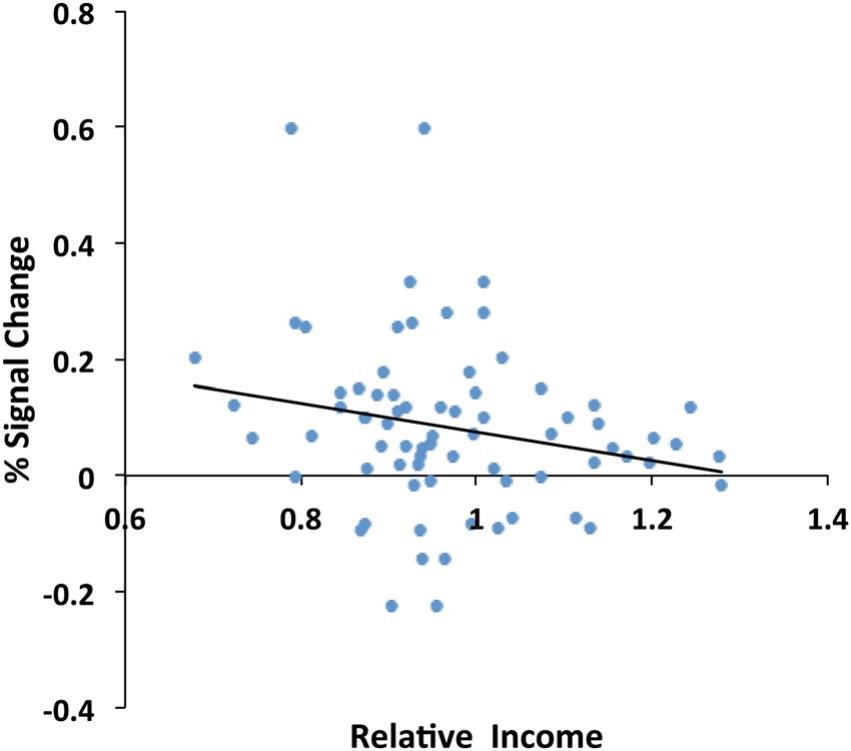
Activation in the caudate nucleus was correlated with expected reward. Activation of the caudate nucleus (14, 10, 8; FWE-corrected *p* = 0.029 for multiple comparisons across the whole brain; *k* = 13) was negatively correlated with relative income (*r* = −0.21, *p* = 0.019) when choosing an equality rule behind the VoI. Neural data were obtained from the contrast that subtracted the activation evident when drawing the lottery from that observed upon a redistributive decision. The relative income was calculated by dividing the participant’s total income by that of the partners. Total income was neither realized nor revealed until the end of the game; thus, the relative income was regarded as an expected reward.

Activation of the caudate nucleus was correlated with the relative income (participant’s total income/partners’ total income; see Methods) when the equality decision was made behind the VoI (*r* = −0.21, *p* = 0.019; Fig. 6). While the decomposition analysis showed that the caudate nucleus was activated by equality decisions alone, a negative correlation was observed only when making equality decisions behind the VoI. The caudate nucleus was more strongly activated by an equality decision when a participant was playing against the odds (i.e., earning a lower income than the other two players). Since the total income was accumulated from the earnings from all rounds and thus realized only at the end of play, the negative correlations indicate that stronger activation was associated with participants’ expectation that their gain would be lower than their partners’. A neural response in the caudate nucleus may reflect an expected reward, i.e., expectation of a reward that the participant is in fact at risk of losing against others.

## Discussion

To explore the neural correlates of equality, our experiment simulated a real income redistribution problem, to which the philosophical debate is closely linked. The VoI places general constraints on selfishness and prevents living creatures from indulging only in activities directed towards self-interest^20^. An individual may invest in fairness in a society in which the worst-off individuals are cared for equally or may choose to reduce their own risk in a worst-off situation. In the former situation, one extends consideration to others but, in the latter, confines it to oneself. Our results imply that an equality decision made behind the VoI cannot be reduced to the more generic systems that underlie decision-making under risk. These apparently distinct motivators at the behavioural level may share the fundamental processes necessary to project one’s self into situations beyond immediate experiences. The neural correlate may distinguish two parallel processes of mental projection in social contexts. One such process is that of projecting one’s self into the perspectives and situations of others, whereas the other is simulating a social situation that has not been experienced but may occur in the future.

Equality decisions behind the VoI but selfless decisions without the VoI activated the dACC and the caudate nucleus. Activation in these regions was stronger when income disclosure decreased the participants’ affection towards their partners with whom they had become favourable towards during game play, indicating that these regions were implicated in the attitude of leaning towards accordance with others. Previous studies have reported observations that support our results in both regions. ACC activation has been considered to be broadly equated with empathy^21, 22^ and, in particular, has been associated with a bias towards accordance and a decision to meet other’s expectations as a result of understating others’ behaviour and mental states. Cooperation to honour other’s trust in a trust game has been associated with activation of the dACC^23^. Recently, the dACC has been linked to a conforming attitude that tends to change one’s beliefs and behaviours to match those of others in a group and those of society at large^24–26^. The caudate nucleus is a component of the reward-related circuit^27^ and is also associated with social rewards derived from general learning about others^28^, trustworthiness, and reciprocity with others^29^ as well as with social risk, that is, a violation of social norms^30, 31^. A recent meta-analysis of the ultimatum game reported that robust activation of both the dACC and caudate nucleus was associated with rejection of unfair offers regardless of one’s own gain, i.e., a fair decision that conformed with that of reciprocal individuals^32^. Using the affective attitude measure, our experiment indicated that the dACC and caudate nucleus were associated with making socially conforming decisions on distributive equality.

Whereas activation of the dACC depended on context with and without the VoI, the caudate nucleus was specifically activated by an equality decision alone. Moreover, with the uncertainty of the VoI, activation in the caudate nucleus represented the expected loss of a reward against others. In this regard, activity in the caudate nucleus may represent surveillance of one’s own payoff in comparison with that of others and thus may be associated with the calculation of self-prospects in the social context. This result is consistent with a recent report that this region was implicated in tracking rewards and responded sensitively to both advantageous and disadvantageous inequality^33^. The caudate nucleus defines and seeks reward contingent on on-going behaviour^34, 35^. Previous studies have shown that the caudate nucleus is involved in reward expectation and risk in general^36^ and in defining both social and monetary rewards in particular^37, 38^.

Together, the neural correlates indicate that the human pursuance of equality may be underpinned by the consideration of others and the prospective calculation of one’s own reward. These disparate presentations of motivations may arise from mental processes that allow for the projection of oneself into alternative situations and thus may share relevant neural circuits. Projecting oneself into a future situation for prospective calculation may involve a cognitive process that is similar to that by which one adopts the perspective of another and infers their feelings and mind set. A recent development in the study of brain activity has linked mental projection to another time or place with the perspectives of others in general^14, 39, 40^. A meta-analysis of fMRI and positron emission experiments found that the brain region linked to prospection or self-projection into a future situation overlapped with a default mode network that involves mental simulation of interactions with others or the theory of mind^14^. More generally, a recent review^15^ raised the possibility that mental simulation mechanisms involved in empathizing with others also underlie the temporal (dis)counting choice of one’s own reward. Developmental studies have long observed a positive relationship between a better performance in theory-of-mind tasks and patience for a delayed reward^41, 42^. The caudate nucleus^43–45^ and the ACC^46, 47^ have also been shown to respond as strongly to a close other’s reward as to one’s own reward. These regions might be involved in simulating oneself as sharing the perspective of others and projecting oneself into a future situation.

In behavioural studies, a consideration of others and a calculation for one’s prospects has been considered opposing motivational forces. The neural correlates of equality suggest that an identical process might fundamentally underpin mental projection, that is, viewing the world from the perspectives of others and simulating one’s own situation in the future. Consequently, the human pursuance of equality in real society might hinge on the mental capacity to project oneself into situations that are distinct from the immediate experience.

## Methods

### Subjects

A total of 33 healthy volunteers (6 females and 27 males with a mean age ± standard deviation of 21.06 ± 2.18 years) recruited from university campuses were screened to exclude those inappropriate for magnetic resonance scanning. All participants were neurologically normal, strongly right-handed^48^, and complied with the movement restrictions during the fMRI sessions. They completed a computer tutorial prior to scanning that fully explained the procedure and were informed that their final reward would be proportional to the total sum of their payoffs from the games. Using a post-scanning questionnaire, we confirmed that all 33 participants believed they had been playing with actual humans. All experimental procedures were performed in accordance with the guidelines set by the ethics committees of the University of Tokyo and Tamagawa University and were approved by these committees. Written informed consent was obtained from all subjects prior to participation in the study, and all subjects were compensated for their participation.

### Procedure and design

All participants were photographed and told that their photos would be shown to two partners in another room who would participate in the games with them. At the beginning of each game, each participant was shown photographs of their partners. Each participant was told that she belonged to a hypothetical society in which three income classes (high, middle, and low) each contained the same number of people. Each of the three classes was randomly assigned to the three players (the participant and two other same-sex partners) from one round to the next. Each participant was aware of only her own income class and was required to choose one among three redistribution rules: preserving the status quo *inequality*, advancing *intermediate* redistribution, or achieving absolute *equality*. During the games, these rules were called rule 1, rule 2, and rule 3, respectively, without giving a bias to the decision. To imitate a real redistribution situation in society, changing the status quo was assumed to have costs. Based on the results of a previous behavioural experiment, we set 25% of the amount of transferred income as the cost to be incurred. Thus, an equality rule would incur the largest transfer cost, and an inequality rule would have no cost. Figure 1A presents the resulting distribution and costs along with the three rules.

The experiment distinguished between two conditions of individual-level decisions: whether the income classes were unknown (VoI) or divulged (Informed) when the participant decided on a redistribution rule. Under the VoI condition, the income class was hidden and thus did not affect the participant’s decision. When the participants were informed of their class, the experimental conditions were further distinguished by the three classes (high, middle, and low).

We distinguished four experimental conditions (VoI, high, middle, and low; Fig. 1B). The meaning of choosing the same rules differed between these conditions: under a VoI condition, a decision favouring the *equality* rule was considered a fair decision involving others or a risk-aversive decision on behalf of oneself, while a non-equality decision favouring the *inequality* or *intermediate* rules was considered an unfair or risk-seeking decision. In an Informed condition of a high (low) income class, *in*e*quality* (*equality*) would be a selfish choice, while *intermediate* or *equality* (*inequality*) rules would be a selfless decision. In the assignment of a middle-income class, all rules involved the same payoff; thus, participants were neutral across rules.

In each round of the game, each participant was required to choose one of three redistribution rules (Fig. 1C; red-boxed screen). After making an individual decision, the participant was told her earnings as a result of the society-level decision, and earnings from each round accumulated into a total income that was revealed at the end of the game. Participants were told that the total incomes determined the final payment for completing the experiment.

We explained to all participants that society-level decisions were made by voting and were decided at random when the votes were split. We also used a dictatorship condition to control for voting (i.e., to examine whether strategic voting was in play): participants were assigned a dictatorship to make a society-level decision in approximately one-third of the rounds, whereas their partners decided a society-level rule as a dictator in the remaining rounds (see Supplementary Information for more detailed procedures). When the dictatorship condition was included as a control, all participants played in four separate game sessions (4 = 2 [VoI/Informed] × 2 [Voting/Dictatorship]), in random order. In repeated rounds of each session, the participant played in all classes (high, middle, and low), which were assigned randomly, and in each class assignment, the participant experienced all society-level outcomes (inequality, intermediate, and equality) in a random order, except when decided as a dictator. We used repeated games since we confirmed in a previous behavioural experiment that neither the decision nor result in the previous round significantly influenced the decision. The reported results did not differ when the voting and dictatorship conditions varied and were thus presented without distinguishing between these conditions.

Additionally, we randomly inserted a lottery game with the same format within each session. In this lottery, participants chose one of three payoff sets that were equivalent to the redistributions resulting from the three rules (Fig. 1C; red-boxed screen). They were then told whether a high, middle, or low payoff would be given. The protocol for the lottery games was similar to the redistribution game under the VoI condition, except that the lottery payoff was not linked to the income class assignment, and the lottery result affected only the participant’s own payoff (see Supplementary Information for detailed procedures).

### Indicators: Affective changes and expected reward

We investigated the attitudinal and behavioural indicators of subjects after they played the game to examine whether these subjective values were correlated with brain activation.

We measured each participant’s affective attitude towards others with a feeling thermometer. Each participant was asked to rate how much they (dis)liked each of the other players on a thermometric scale, on which 0° meant “least favourable”, 100° meant “most favourable”, and 50° meant “neutral”. Thermometric scales have been widely used in the social sciences^49, 50^ and have been employed in fMRI studies^29, 51^. Before and after playing each session, participants were shown photographs of their partners and were asked to rate their feelings (Affective Feeling I and II); they were also asked to rate their feelings again (Affective Feeling III) after the total income of all players was revealed. We calculated the *change in affection after playing* by subtracting Affective Feeling I from Affective Feeling II and the change in affection *after income disclosure* by subtracting Affective Feeling II from Affective Feeling III.

To control for the effect of material vantage on the affective feeling, we focused on the cases in which the total income was higher than others and examined the relationship between affective changes after playing and after income disclosure. We distinguished those with affective changes that were unexpected and expected from the material vantage. Based on the comparison of the contrasting pattern, i.e., going up (down) and up (down), in the ratings of Affective Feelings I, II, and III, we regarded them as the ones to prefer *accordance* and *non*-*accordance* with others, respectively.

Among the seventy-eight cases with participants who earned a higher income than their partners’ in the sessions where equality/selfless decisions (that were included in the neural analysis) were observed, we excluded twenty-four cases that included participants who did not change their affective rating after income disclosure (0 = Affective Feeling III – Affective Feeling II), and four contradictory cases where the participants revealed the *accordance* attitude to one partner but the *non*-*accordance* attitude to the other partner in the same session. In the resulting fifty cases, we found nineteen cases with the *accordance* attitude and thirty-one cases with the *non*-*accordance* attitude (see also Supplementary Information). This categorization was used to examine differential activation in the brain.

We also calculated the relative incomes (participant’s total income/partners’ total income) and explored any correlation thereof with brain activation while making a decision. Since any total income had yet to be realized (and was thus undisclosed) until the end of playing, the relative income was regarded as an expected reward during decision-making that involved prospective calculation for the gain/loss relative to others. For the relative income, we used division rather than the difference because the incomes of the three players varied across sessions due to randomization. We examined the correlation between brain activation and the relative income.

### fMRI acquisition

Functional imaging was conducted using a 3-Tesla Siemens Trio Tim MRI scanner (Erlangen, Germany) at Tamagawa University. For each participant, we acquired whole-brain, T1-weighted, anatomical scans (repetition time (TR), 2,000 ms; echo time (TE), 1.98 ms; flip angle (FA), 10°; field of view (FOV), 256 mm; matrix, 256 × 256; slice thickness, 1.0 mm; 192 sagittal slices) and gradient echo, T2-weighted, echo planar images (EPI) with BOLD contrast (TR, 2,500 ms; TE, 25 ms; FA, 90°; FOV, 192 mm; matrix, 64 × 64; slice thickness, 3.0 mm; 42 oblique axial slices). We used a tilted acquisition sequence at 30° to the AC-PC line to prevent signal loss in the medial orbitofrontal cortex. Each brain volume comprised 42 axial slices of 3-mm thickness and 3-mm in-plane resolution.

### fMRI analysis

We subtracted the contrast images in the lottery game (3 s, Fig. 1C; red-boxed screen in the middle) from the ones during the decision stage in the redistribution game (3 s, Fig. 1C; red-boxed screen on the top and bottom) in each session. Using this contrast, we controlled for the effect of risk consideration on one’s own payoff in relation to the lottery decision. We offset the effect of self-risk consideration and focused on the redistribution-specific activation that specifically reflected a decision involving redistribution to others. A general linear model (GLM) was used to estimate the parameters for each experimental condition along with a participant’s decision on a redistribution rule, namely, *inequality*, *intermediate*, and *equality*, under the four different experimental conditions (VoI, high, middle, and low) in each session. The GLM also included six additional regressors of no interest to model head movement.

In the second level of the analysis, the individual-level design matrix of the 33 subjects was used to estimate, in reference to the experimental conditions (VoI, high, middle, and low), participant decisions on the redistribution rules (*inequality*, *intermediate*, and *equality*) and the society-level decision methods that did not affect the reported results. We used factorial analysis to explore brain data of a homogenous group of 33 subjects who played all sessions in a randomized order. As described in a previous study^29^, this factorial design allowed us to identify activation that was associated with the randomly assigned conditions. We applied a threshold of *p* < 0.05, corrected for FWE, for multiple comparisons across the whole brain, combined with a cluster-size threshold of 10 voxels on an FWE-corrected *p* < 0.05 *t*-map for multiple comparisons across the whole brain. For brain activation, we calculated the percentage of signal changes using rfxplot (revision 19)^52^. Activated regions were labelled anatomically using Neurosynth (www.neurosynth.org) and located visually using an anatomic atlas^53^. All *x*, *y*, and *z* coordinates are reported in Montreal Neurological Institute space.

### Decomposition of the interaction effects

Analysis of the interaction effects in high-dimensional data involves a methodological challenge. In fMRI analysis, in particular, estimation of the effects depends on the baseline activity level; thus, interpretation of the empirical findings is also influenced by the choice of baseline^54^. To solve this problem, we decomposed the effects of the VoI and the decision of the equality rule on activation using a newly developed method^55^. More specifically, we calculated an effect whose relative magnitude did not depend on the choice of baseline condition for the higher-order interaction. We examined the invariant effect of each factor, especially the VoI and equality decision, on brain activation (see Supplementary Information [2] for more details).

We calculated an invariant interaction effect, *π*, the average marginal treatment interaction effect based on *τ*, the average treatment combination effect, and *ψ*, the average marginal treatment effect, as follows:

$$\begin{array}{ll} & \pi ({\rm{VoI}},{\rm{Equality}};{\rm{High}},{\rm{Inequality}})\\ = & \tau ({\rm{VoI}},{\rm{Equality}};{\rm{High}},{\rm{Inequality}})-\psi ({\rm{VoI}};{\rm{High}})-\psi ({\rm{Equality}};{\rm{Inequality}})\\  & \tau ({\rm{VoI}},{\rm{Equality}};{\rm{High}},{\rm{Inequality}})\\ = & \bar{Y}({\rm{VoI}},{\rm{Equality}})-\bar{Y}({\rm{High}},{\rm{Ineuality}})\\  & \psi ({\rm{VoI}};{\rm{High}})\\ = & \{\bar{Y}({\rm{VoI}};{\rm{Inequality}})-\,\bar{Y}({\rm{High}};{\rm{Inequality}})\}\cdot {\rm{P}}({\rm{Inequality}})\\  & +\,\{\bar{Y}({\rm{VoI}};{\rm{Intermediate}})-\bar{Y}({\rm{High}};{\rm{Intermediate}})\}\cdot {\rm{P}}({\rm{Intermediate}})\\  & +\,\{\bar{Y}(\mathrm{VoI};\mathrm{Equaltiy})-\bar{Y}(\mathrm{High};\mathrm{Equality})\}\cdot {\rm{P}}({\rm{Equality}})\\  & \psi (\mathrm{Equality};\mathrm{Inequality})\\ = & \{\bar{Y}(\mathrm{Equality};\mathrm{High})-\bar{Y}(\mathrm{Inequality},\mathrm{High})\}\cdot {\rm{P}}({\rm{High}})\\  & +\,\{\bar{Y}(\mathrm{Equality};\mathrm{Low})-\bar{Y}(\mathrm{Inequality};\mathrm{Low})\}\cdot {\rm{P}}({\rm{Low}})\\  & +\,\{\bar{Y}(\mathrm{Equaltiy},\mathrm{VoI})-\bar{Y}(\mathrm{Inequality},\mathrm{VoI})\}\cdot {\rm{P}}({\rm{VoI}})\end{array}$$

In these equations, $\bar{Y}$ is the mean value of the percent signal change that corresponds to each combination of rule choices and experimental conditions. The differences in $\bar{Y}$ are weighted by the observed frequency of rule choices or conditions (P) in calculating the average marginal treatment effects. Intuitively, the decomposed interaction effect (*π*) can be calculated by subtracting the effect of each variable (*ψ*) from the combined effect of two variables (*τ*).

### Correlation between attitudinal and behavioural indicators and brain activity

We investigated the attitudinal and behavioural indicators of subjects after they played the game and examined whether these subjective values were correlated with the redistribution-specific activation identified after correcting for multiple comparisons across the whole brain. This procedure enabled us to avoid the problems of reverse inference^16, 17^ and circular analysis^56^ when we inferred the cognitive process behind the activation.

We examined differential activation between the accordance and non-accordance attitudes with others. We also examined whether brain activation was correlated with the relative income. Since the neural results include the same participants’ data in different sessions (note that we applied the factorial analysis to the neural data), we further confirmed whether the results did not depend on the assumption on the independence of the data. We applied the bootstrapping analysis based on the assumption that the same participant’s data were not independent, and confirmed that all the results were still above the statistical threshold (*p* < 0.05).

### Data availability

The datasets generated or analysed during the current study are available from the corresponding author on reasonable request.

## Electronic supplementary material

Supplementary Information
