# Supplementary material for: Human pursuance of equality hinges on mental processes of projecting oneself into the perspectives of others and into future situations
Source: Sci Rep. 2017 Jul 19;7:5878. doi: 10.1038/s41598-017-05469-9 (PMC5517530; doi:10.1038/s41598-017-05469-9)
Supplement: Supplementary file 1 — Supplementary Information [file 41598_2017_5469_MOESM1_ESM.pdf]

Human pursuance of equality hinges on mental processes of projecting oneself into the perspectives of others and into future situations

Hirofumi Takesue[1†], Carlos M. Miyauchi[1,3†], Shiro Sakaiya[4], Hongwei Fan[5], Tetsuya Matsuda[3] and Junko Kato[1,2]*

[1] Graduate School of Law and Politics, the University of Tokyo

[2] Institute for Diversity and Adaptation of Human Mind, the University of Tokyo

[3] Tamagawa University Brain Science Institute

[4] Graduate School of Social Sciences, Tokyo Metropolitan University

[5] Oracle (China) Software Systems Co., Ltd.

Correspondence:

Junko Kato, The University of Tokyo, 7-3-1 Hongo, Bunkyo, Tokyo, 113-0033, Japan

Correspondence to e-mail: *katoj@j.u-tokyo.ac.jp*

[†]These authors have contributed equally to this work.

Supplementary Information

[1] Task inside fMRI

An experimental framework of redistribution games was devised, based on the result of behavioral experiments (3 players x 45 groups = 135 subjects) in which we compared two different sets of redistribution rules and two different proportions of costs. If we compared the level of (in)equality of three redistribution rules by using the Gini coefficients that represent absolute equality by 0 and absolute inequality by 1, Rules 1, 2, and 3 have the value of 0.36, 0.19, and 0, respectively. The level of equality of Rule 1 was set close to 0.32, the average of thirty-four member countries of the Organisation for Economic Co-operation and Development, as well as 0.34, the value assigned to the Japanese society as of 2012. The level of equality of Rule 2 was the same as the level of Sweden in the early 1980s when the country achieved its highest level of equality. We devised the experimental framework to examine brain activity when participants were sensitively responding to redistribution rules from absolute equality to a moderate level of inequality.

Participants played four separate sessions in a randomized order. The redistribution game was repeated so that a participant played in all (high/middle/low) classes that were randomly assigned and, in each class assignment, experienced all society-level outcomes (inequality / intermediate / equality) in a random order except when she decided it as a dictator. Therefore, the number of rounds varied across sessions, from 12 to 40, i.e., from the VoI to informed sessions, respectively.

[2] Decomposition of ANOVA effects

One important methodological challenge of the fMRI data was the analysis of the interaction effects in the high-dimensional data. The estimation of the effects depends on the baseline activity level; thus the interpretation of the empirical findings was also influenced by the choice of the baseline.[58] To solve this problem, we have used the method developed by Imai and Egami[59] and examined the invariant effect of each factor, especially the VoI and Equality decision, on brain activation. Imai and Egami define the concept of the *marginal treatment interaction effect*, whose relative magnitude does not depend on the choice of a baseline condition for higher-order interaction. Then, they developed a method by which we can calculate the effect of the interaction in the high-dimensional data so as to be invariant to the choice of a baseline category. The details about the methodological information and the software for the analysis are found

at http://imai.princeton.edu/research/int.html.

[3] Categorization of the attitudes with *accordance* and *non-accordance* with others

Among one hundred and thirty-two cases (132 cases = 33 participants x 4 sessions x 2 partners) we found seventy-eight cases when participants earned higher income than partners' in the sessions that observed an equality/selfless decisions (and thus included in the neural analysis), we excluded twenty-four cases when participants did not change the affective rating after income disclosure (0 = Affective Feeling III – Affective Feeling II) and four contradictory cases when two participants revealed *accordance* attitude to one partner but *non-accordance* attitude to other partner in the same session. In resulting fifty cases, we found nineteen cases with the *accordance* attitude and thirty-one cases with *non-accordance* attitude. Note that the same participants' data were included more than once if they revealed the same attitude in different sessions. When they showed the same attitude to different partners in the same session we averaged the changes between different partners. Since the data of seven participants were included twice in *accordance* and fourteen in *non-accordance,* we counted twelve participants (female = 4) who ever revealed the *accordance* attitude and seventeen participants (female = 2) who ever revealed the *non-accordance* attitude. Six participants (female =1) ever revealed the both attitude and thus twenty-three participants' data were included in the analysis.

Supplementary Table 1 The results of redistibution games

A Proportion and total number of trials in which inequality, intermediate and equality rules were chosen in each class assignment

	Informed			VoI
	High	Middle	Low	Veil of Ignorance
inequality	80% (568)	9% (96)	3% (20)	48% 370
intermediate	14% (98)	23% (263)	7% (50)	24% 185
equality	6% (40)	68% (768)	90% (636)	28% 211
	100% (706)	100% (1127)	100% (706)	100% 766

B Proportion and number of subjects who (ever) chose inequality, intermediate and equality rules in each class assignment

	Informed			VoI
	High	Middle	Low	Veil of Ignorance
inequality	100% (33)	61% (20)	33% (11)	85% (28)
intermediate	55% (18)	88% (29)	42% (14)	70% (23)
equality	39% (13)	97% (32)	100% (33)	76% (25)

[shaded] = selfless / equality decision

Supplementary Table 2 : Attitude toward Risk

A. The Results in All

	RiskSeeking in Lottery	RiskAverse in Lottery	Intermediate in Lottery	Sum
RiskSeeking in Redistribution	26	0	2	28
RiskAverse in Redistribution	5	8	2	15
Intermediate in Redistribution	7	2	14	23
Sum	38	10	18	66

When participants chose the inequality rule more than 60% of the time we define they were more risk seeking. A risk-averse tendency was observed among 15% of those who chose the equality rule more than 60% of the time. Overall, 27% were found in between. In the risk-seeking group, 26% (in dictatorship) to 47% (in voting) shifted away from risk-seeking in the redistributive decision behind the VoI, whereas the risk-averse groups in the lottery also avoided risk in the redistributive decision.

Supplementary Table 3

ANOVA Result [Dictataor/Vote] x [High/Middle/Low/Veil of Ignorance] x [Inequality/Intermediate/Equality]

	Region	BA	MNI coordinates			k	FWEcorrectedF	F(1,444)		Z
			x	y	z					
Main effect of Dictator/Vote	Inferior Parietal L	BA40	−52	−48	42	646	0.002	27.84		5.06
			−36	−60	−34	504	0.002	27.53		5.03
			26	−74	−28	384	0.018	22.66		4.55
	Inferior Parietal L	BA40	50	−54	44	422	0.039	20.87		4.36
	rDLPFC	BA8	2	34	44	338	0.067	19.60		4.22
									t(444)	
Positive effect of Vote > Dictator	Inferior Parietal L	BA40	−52	−48	42	137	0.001		5.28	5.19
			−36	−60	−34	177	0.001		5.25	5.17
			26	−74	−28	35	0.009		4.76	4.70
	Inferior Parietal L	BA40	50	−54	44	17	0.020		4.57	4.51
	rDLPFC	BA8	2	34	44	13	0.034		4.43	4.38
								F(3,444)		
Main effect of HMLVoI	insula	BA13/44	−42	6	4	303	0.011	10.39		4.70
	Inferior Parietal L	BA45/46/47	−50	36	0	143	0.045	9.23		4.37
									t(444)	
Positive effect of L > VoI	insula	BA13/44	−42	8	4	6	0.018		4.59	4.54
		BA47	28	26	−8	371	0.099		4.13	4.08
Positive effect of H > VoI	Inferior Parietal L	BA45/46/47	−50	36	0	70	0.001		5.23	5.15
		BA6/43	62	−4	12	13	0.017		4.60	4.54
								F(6,444)		
Interaction of HMLVoI x Rules	anterior insula		−32	26	4	167	0.006	6.86		4.87
	lDLPFC	BA9	−40	10	30	275	0.019	6.34		4.61
	dACC	BA32	4	16	38	628	0.061	5.81		4.32
	caudate nucleus		14	10	8	188	0.071	5.73		4.28
		BA18/19	16	−48	0	223	0.07	5.51		4.16
Positive Interaction Selfless/Equality (VoI) > Selfish/Non-equality(VoI)										
	anterior insula		−32	26	4	165	0.001		5.31	5.23
	lDLPFC	BA9°	−40	10	30	257	0.001		5.45	5.36
	dACC	BA32	4	16	38	116	0.006		4.87	4.80
	caudate nucleus		14	10	8	13	0.029		4.47	4.41

sub-maxima within in a cluster °

FWE corrected p < 0.1 for multiple comparisons across the whole brain for main effect/interaction : the number of k on uncorrected F-map

FWE corrected p < 0.05 for for multiple comparisons across the whole brain positive effect/positive interaction : the number of k on FWE corrected p < 0.05 t-map

Selfless = High(Intermediate/Equality) : Low(Equality/Intermediate)

Selfish = High(Inequality) : Low(Equality)

The BA9 and anterior insular were excluded from the further analysis since activation behind the VoI was not differentiated by equality and non-equality decision.

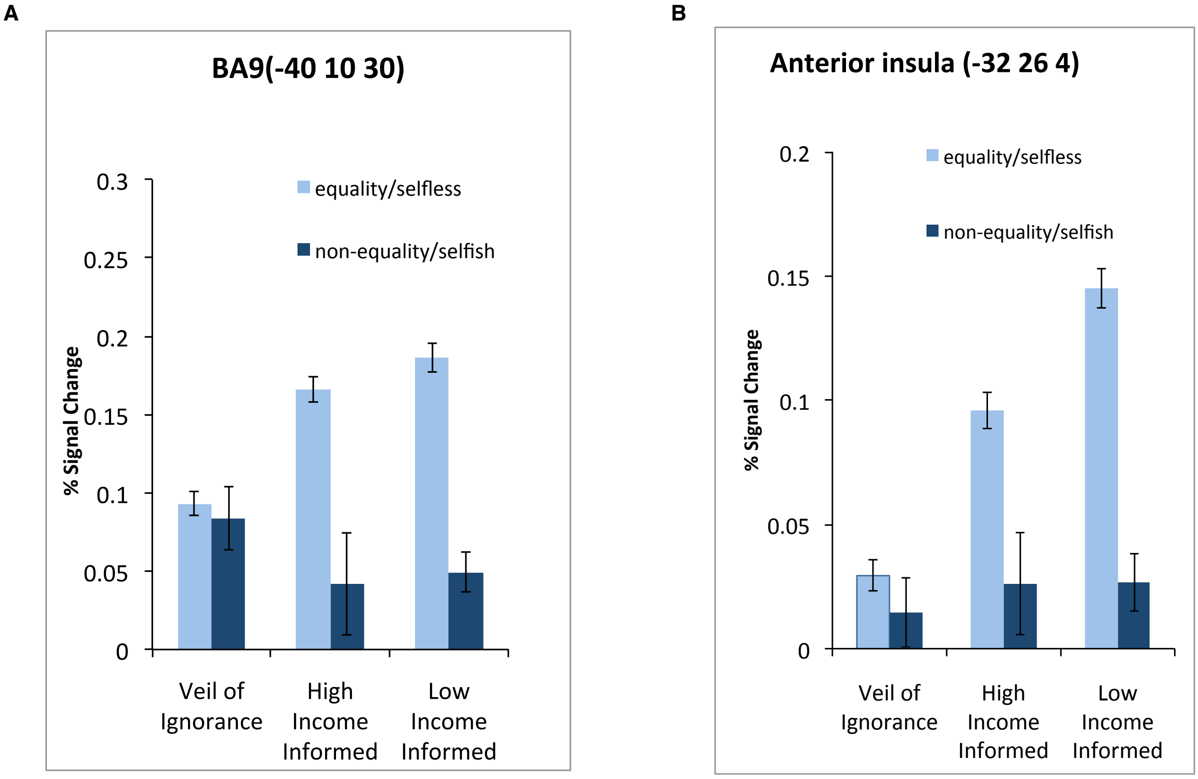

Supplementary Figure 1

The BA9 and anterior insular also revealed an interaction (see Supplementary Table 3) but were excluded from the main result since activation behind the VoI was not significantly differentiated by equality and non-equality decision.

A. The coordinates in BA9 and anterior insult were included in the ANOVA result but were not included in the analysis. Both equality and inequality rules activated these regions in the VoI conditions.

B. anterior insular (-32, 26, 4), FWE corrected p = 0.001, k = 165 (FWE corrected p < 0.05)

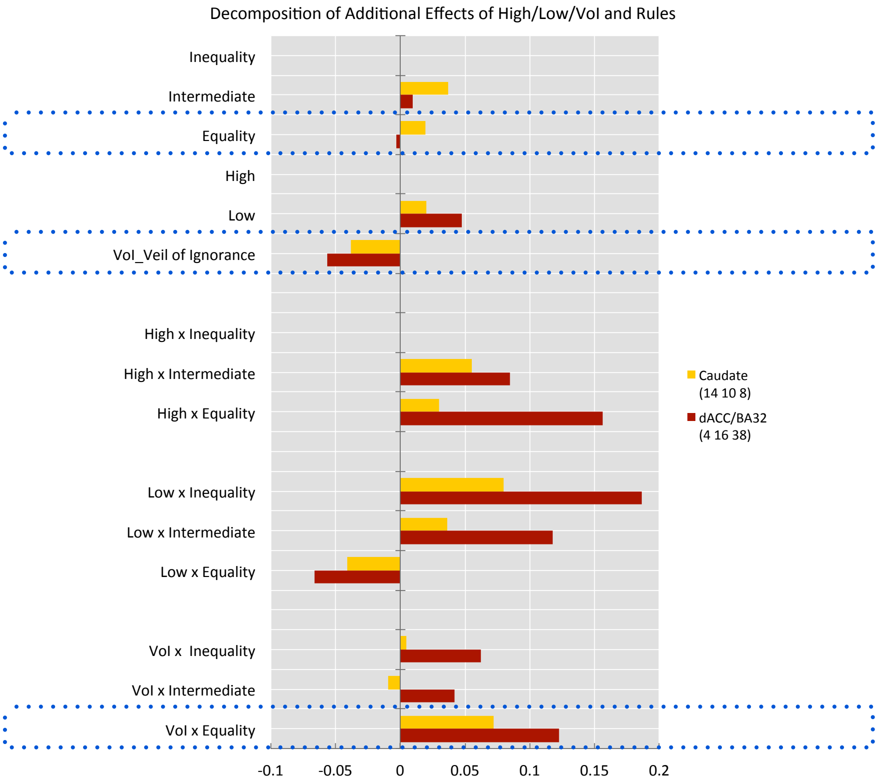

Supplementary Figure 2

Average Marginal Treatment Interaction Effects(AMTIEs) represent additional effect of each factor and is calculated based on % signal change.

The base line is set at activation level associated with High Inequality.

Compared with activation associated with the lottery decision, the VoI condition specifically reduced activation in the dACC as well as in the caudate nucleus but neither high- nor low-income class conditions.
